# Developing an Approach to Legal and Ethical Risks of Clinical Use of Biomarker Testing for Alzheimer’s disease

**DOI:** 10.1002/alz70861_110877

**Published:** 2025-12-23

**Authors:** Drew Blasco

**Affiliations:** ^1^ University of Nevada, Las Vegas, Las Vegas, NV USA

## Abstract

**Background:**

The 2024 Clinical Guidelines and Staging Framework (Jack et al., 2024) further advances the integration of biomarker testing for clinical use. However, legal and ethical challenges persist regarding the implementation of biomarker testing, including the potential risk of discrimination following such testing. In December 2024, the Alzheimer’s Association science and public policy teams convened with a selection of experts (including a pre‐established workgroup) to discuss the rapidly evolving landscape of biomarkers for clinical use, with a focus on blood‐based biomarkers (BBM). The Association charged meeting participants with evaluating disclosure of clinical biomarkers to patients and to consider potential consequences, including discrimination (e.g., insurance, employment, housing, healthcare). The Workgroup identified a need to collect evidence that will support future policy development and positions that aim to mitigate potential discrimination risks. Two foundation research needs were identified: (1) a better understanding of what evidence currently exists, including the status of perceived, observed, and possible legal risks and (2) prioritization of the legal protections needed for patients in Stages 1 (asymptomatic, biomarker evidence) & 2 (transitional decline) within updated staging framework.

To meet these identified needs, panelists will discuss ongoing work to identify and describe the perceived, observed, and possible threat of discrimination based on disclosure of one’s biomarker status for AD patients. Panelists will first report on prior work that informs a need to understand discrimination and other legal/ethical risks. We will then report on our approach to bridge gaps between the existing evidence on these issues and to mitigate discrimination risks due to biomarker testing within a clinical setting. During the panel discussion, panelists will summarize the current state of knowledge regarding the perceived, observed, and possible risk of discrimination based on AD biomarker disclosure in clinical settings, propose an approach to develop a legal framework, and share plans for synthesizing our findings. We will seek engaged feedback from attendees to help prioritize findings and develop approaches for next steps, including a future research agenda and educational products.

**Method:**

N/A

**Result:**

N/A

**Conclusion:**

N/A

